# Obesity Exacerbates Acute Gastrointestinal Injury and Intestinal Barrier Dysfunction in Early-Stage Acute Pancreatitis

**DOI:** 10.5152/tjg.2023.22145

**Published:** 2023-04-01

**Authors:** Qing Huang, Zhe Wu, Yuanyuan Zhang, Yun Wu, Chenchen Shi, Yulan Liu

**Affiliations:** 1 Department of Gastroenterology, Peking University People’s Hospital, Beijing, China; 2 Department of Gastroenterology, Beijing Tsinghua Changgung Hospital, School of Clinical Medicine, Tsinghua University, Beijing, China

**Keywords:** Acute pancreatitis, intestinal barrier, intestinal injury, obesity

## Abstract

**Background:**

The impact of obesity on the severity of acute pancreatitis and subsequent acute gastrointestinal injury remains an important consideration. This study aimed to determine the clinical relationship between obesity and acute gastrointestinal injury in early-stage acute pancreatitis.

**Methods:**

This was a prospective study that enrolled 194 acute pancreatitis patients.

**Results:**

The median body mass index was 26.5 (7.0) kg/m^2^. Considering etiology of acute pancreatitis, 90 patients had gallstones, 48 had hypertriglyceridemia, 36 were alcohol users, and 20 were others. A total of 116 patients had mild acute pancreatitis and the rest had severe acute pancreatitis. The median of bedside index of severity in acute pancreatitis score was 1 (2) and the serum concentration of C-reactive protein was 80.5 (60.0) mg/L. Acute pancreatitis was accompanied by multiple organ dysfunction in 60 cases and by pancreatic necrosis in 34. A total of 52 patients were admitted to intensive care unit. The values of body mass index were higher in patients with severe acute pancreatitis than those with mild acute pancreatitis. A similar trend emerged in patients with hyperlipidemic acute pancreatitis compared to other causes. Body mass index had a positive relationship with bedside index of severity in acute pancreatitis scores. Noticeably, body mass index was statistically raised from gastrointestinal injury grade 1 to grade 4. The values of body mass index also showed relevance with intestinal barrier function evaluated by d-lactate, diamine oxidase, and intestinal fatty acid binding proteins. Furthermore, body mass indexes were statistically higher in patients having adverse outcomes of acute pancreatitis.

**Conclusion:**

This prospective study showed that obesity might contribute to increasing the severity of acute pancreatitis and aggravate subsequent intestinal injury in early-stage acute pancreatitis.

**Main Points**

- Obesity contributed to the disease severity in early-stage acute pancreatitis (AP), presenting as more severe AP (SAP) patients, higher bedside index of severity in acute pancreatitis (BISAP) scores and more adverse outcomes.
- Obese AP patients suffered from worse intestinal function, with higher acute gastrointestinal injury (AGI) scores and increasing intestinal barrier permeability.
- The values of BMI in hyperlipidemic AP patients were higher than those in other AP patients.

## Introduction

Approximately one-fifth of all patients with acute pancreatitis (AP) will develop to severe AP (SAP) characterized by organ dysfunction and pancreatic necrosis with high mortality rate.^1^ Old age, hyperlipemia, obesity, and bedside index of severity in acute pancreatitis (BISAP) or acute physiology and chronic health evaluation II (APACHE II) score would predict the development of SAP. Obesity is a prevalent public health concern over the past few decades.^2^ There is increasing incidence of AP accompanied by high body mass index (BMI).^3^ Several literatures linked obesity factors with SAP, in which BMI was added to traditional score systems resulting in greater diagnostic accuracy.^4,5^ Intestinal dysfunction plays an important role in SAP attributing to bacterial and endotoxin translocation.^6^ Several literatures reported that obesity would damage the intestinal mucosal barrier and change intestinal microbiome.^7^ However, the gastrointestinal function under obesity during the early stage of AP is unknown. The European Society of Intensive Care Medicine developed the definition for gastrointestinal dysfunction in intensive care patients on the basis of available evidence of pathophysiology, which called acute gastrointestinal injury (AGI) grading system.^8^ The aim of this study was to provide further data on AGI and intestinal barrier dysfunction in obese patients during early-stage AP to determine that obesity may exacerbate pancreatitis through intestinal injury.

## Materials and Methods

### Data Source and Study Population

This prospective study included 194 adult AP patients within 24 hours after the onset in gastroenterology and emergency department in Peking University People’s Hospital, a general hospital in Beijing, between January 2017 and December 2019. Acute pancreatitis was diagnosed when at least 2 of the following features occurred: acute persistent severe upper abdominal pain, serum amylase or lipase 3 times greater than the limit, and the AP characters on imaging examinations.^9^ Patients with the following conditions were excluded: (1) other serious systemic diseases, (2) malignant tumors, (3) uncontrolled autoimmune diseases, (4) severe infections, (5) history of gastrointestinal operation, (6) medical history of antibiotic or probiotics in the latest month, and (7) pregnant women.

Blood samples were collected at admission and then stored at −80°C for later use. Levels of d-lactate (DLA), diamine oxidase (DAO), and intestinal fatty acid binding proteins (I-FABP) were assessed by human enzyme-linked immunosorbent assay (ELISA) test kit, which could reflect the intestinal permeability.^10^

### Statement of Ethics and Informed Consent

This study was approved by the ethics committee of Peking University People’s Hospital. All the patients enrolled in the study were well informed and signed the informed consents.

#### Definitions of Severe Acute Pancreatitis and Multiple Organ Dysfunction Syndrome

Majority of patients had mild AP (MAP) with no local or systemic complications, who would recover in 1-2 weeks with a low mortality. Severe acute pancreatitis patients were defined as those who suffered from transient or persistent local complications, like pancreatic parenchymal necrosis, or systemic organ failure.^9^ Patients with moderate SAP and SAP according to the revised Atlanta classification were both classified as SAP in our study. Marshall multiple organ dysfunction (MODS) score system was utilized to assess the function of target organs in AP. The Marshall scoring was acquired at the most severe condition of the patients. The score >2 of an individual organ was supposed to be dysfunction or failure.^11^ More than one organ developed to have dysfunction or failure was defined as MODS.

### Definition of Acute Gastrointestinal Injury

Acute gastrointestinal injury is a dysfunction of the gastrointestinal tract in severe patients due to the primary acute illness. According to the gastrointestinal symptoms, the feeding tolerance, and the influence on other organs, there are 4 grades of severity. Acute gastrointestinal injury grade 1: increased risk of developing gastrointestinal dysfunction, which is a self-limiting condition; AGI grade 2: gastrointestinal dysfunction requiring interventions; AGI grade 3: gastrointestinal dysfunction or failure which cannot be restored with interventions; AGI grade 4: dramatically manifesting life-threatening gastrointestinal dysfunction or failure.^8^ The AGI scoring was evaluated at admission.

### Enzyme-Linked Immunosorbent Assay

Levels of DLA, DAO, and I-FABP in serum were measured using ELISA kits as per manufacturer’s instruction (eBioscience, San Diego, Calif, USA). All reagents and working standards were prepared. A total of 300 µL wash buffer was added to each well and the liquid was then removed. A total of 100 µL of standard and sample was added to each well, which were covered with the adhesive strip provided. Each sample was tested 3 times. A total of 50 µL detection antibody was added to each well. The sample was incubated for 1.5 hours at 37°C. Liquid was removed from each well, which was washed 6 times with 300 µL wash liquid. A total of 100 µL horseradish peroxidase (HRP) avidin was added to each well. The microtiter plate was covered with a new adhesive strip. The sample was incubated for 0.5 hour at 37°C. Liquid was removed from each well, which was washed 6 times with 300 µL wash liquid. A total of 100 µL tetramethylbenzidine (TMB) substrate was added to each well. The sample was incubated for 15 minutes in darkness at 37°C. A total of 100 µL stop solution was added to each well. The optical density of each well was determined within 5 minutes, using a microplate reader (SZJMLY DK-3506) set to 450 nm.

### Statistical Analysis

All the analyses were conducted using Statistical Package for Social Sciences version 26 software (IBM Corp.; Armonk, NY, USA). Measurement data in normal distribution were described by mean and standard deviation. Measurement data in non-normal distribution were described by median and interquartile range. Enumeration data were described by percentage. The Mann–Whitney test was used to compare non-normally distributed continuous variables. Pearson coefficient was used to assess correlations. Z value was used to compare Pearson coefficients. *P* < .05 was considered statistically significant.

## Results

### Clinical Features

A total of 194 AP patients were eligible for our study, whose median of BMI was 26.5 (7.0) kg/m^2^. A total of 121 of 194 were male patients (62.4%), with mean ages of 57.2 ± 16.6 years. All patients were Asian. Considering the etiology of AP, 90 (46.4%) had gallstones, 48 (24.7%) had hypertriglyceridemia, 36 (18.6%) were alcohol users, 10 (5.2%) had post-endoscopic retrograde cholangiopancreatography (ERCP), and 10 (5.2%) were others. A total of 116 patients (59.8%) were diagnosed with MAP and the rest were diagnosed with SAP. The BISAP score^12^ was 1 (2) and the serum concentration of C-reactive protein (CRP) was 80.5 (60.0) mg/L. Acute pancreatitis was complicated with MODS in 60 of 194 cases (30.9%) and by pancreatic necrosis in 34 (17.5%). The length of hospital stay was 12 (7.5) days. A total of 52 (26.8%) patients were transferred to intensive care unit (ICU), who stayed in ICU for 3.5 (5) days. Furthermore, the mortality of AP patients was 5.2% (Table 1).

### Relating Body Mass Index and Acute Pancreatitis

**Figure 1.**
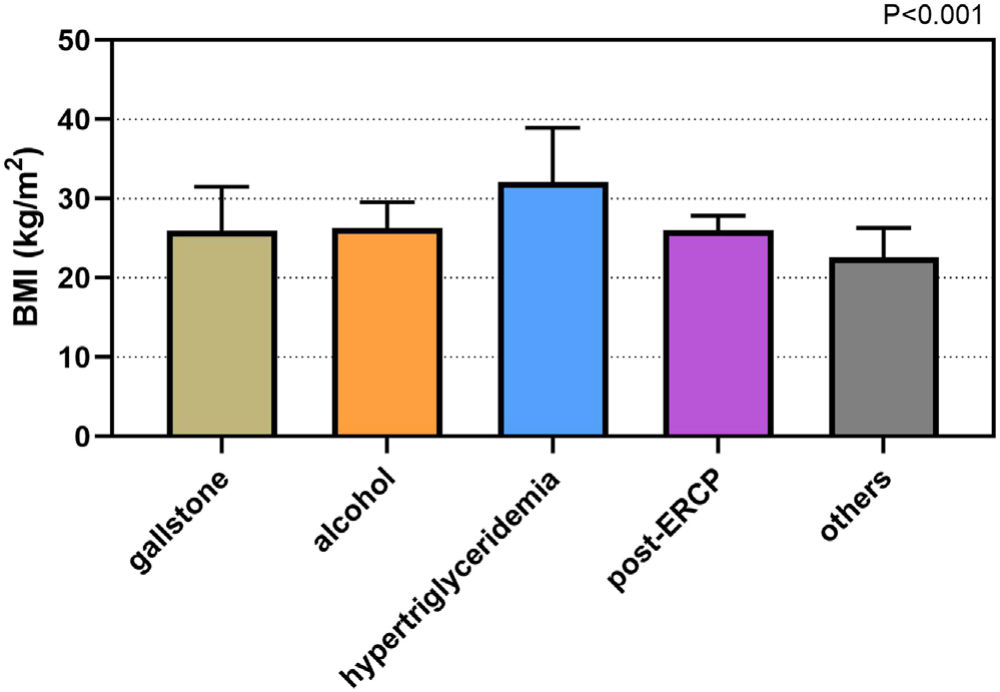
Values of BMI in different groups of AP etiology. The values of BMI in hyperlipidemic AP patients were statistically higher than those in other AP patients. BMI, body mass index; AP, acute pancreatitis.

**Figure 2.**
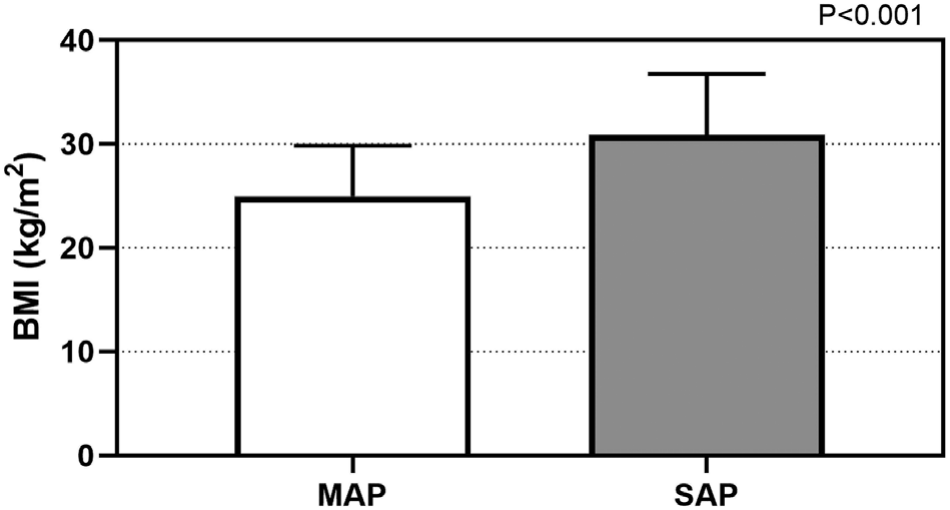
Values of BMI in the MAP and SAP groups. The values of BMI in SAP patients were statistically higher than those in MAP patients. BMI, body mass index; AP, acute pancreatitis; MAP, mild acute pancreatitis; SAP, severe acute pancreatitis.

**Figure 3.**
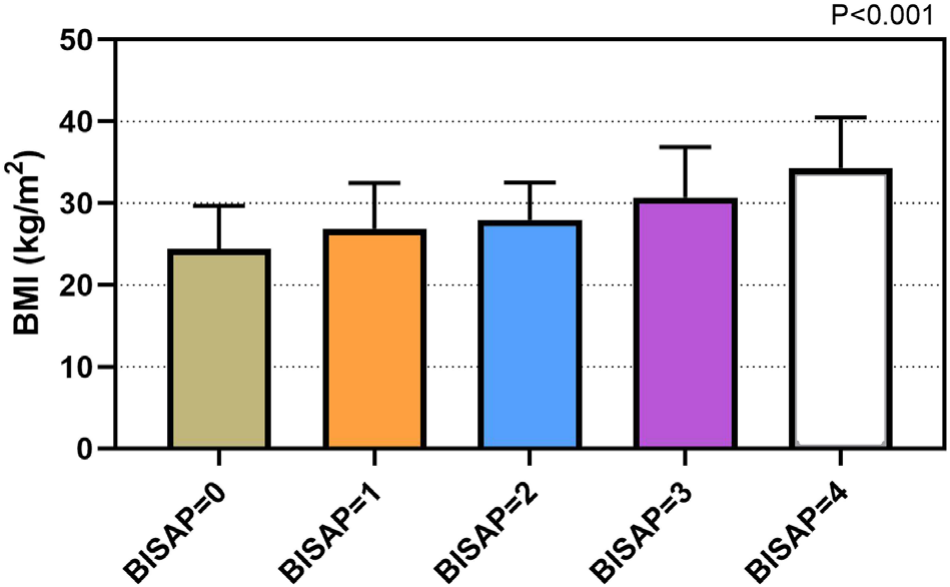
Values of BMI in different groups of BISAP score. The values of BMI were significantly different according to BISAP score system, which increased respectively from BISAP 0 to 4. BMI, body mass index; BISAP, bedside index of severity in acute pancreatitis.

Considering etiology of AP, the values of BMI were statistically different (*P* < .001). The levels of BMI were 24.8 (6.7) kg/m^2^ in gallstones group, 31.1 (8.0) kg/m^2^ in hypertriglyceridemia group, 27.6 (5.4) kg/m^2^ in alcohol group, 26.5 (2.9) kg/m^2^ in post-ERCP group, and 24.4 (6.3) kg/m^2^ in others group, respectively. The values of BMI in hyperlipidemic AP patients were statistically higher than those in other AP patients (*P *< .001) (Figure 1). Similarly, the values of BMI were higher in patients suffering from SAP (30.2 (9.7) kg/m^2^) when compared to those suffering from MAP (25.4 (7.5) kg/m^2^), *P *< .001 (Figure 2). Values of BMI were significantly different according to BISAP score system (*P *< .001), which from score 0 to 4 were 24.3 (7.0) kg/m^2^, 26.5 (8.4) kg/m^2^, 27.8 (6.5) kg/m^2^, 30.2 (8.4) kg/m^2^, and 30.3 (19.0) kg/m^2^, respectively (Figure 3).

### Body Mass Index and Acute Pancreatitis Associated Gastrointestinal Dysfunction

**Figure 4.**
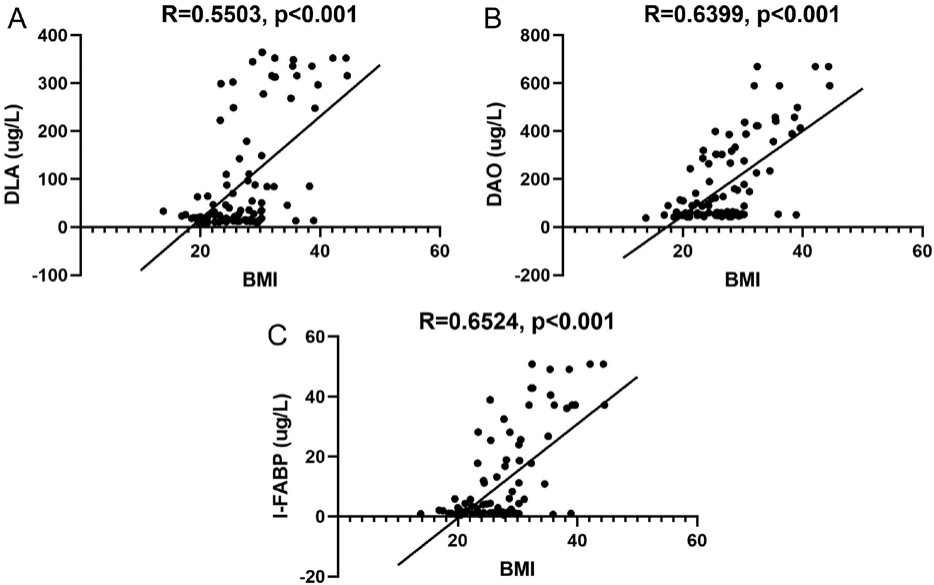
Correlation between BMI and intestinal barrier permeability. (A) Correlation between BMI and DLA; (B) correlation between BMI and DAO; (C) correlation between BMI and I-FABP. There was a moderate correlation between BMI and the serum levels of DLA, DAO, and I-FABP. BMI, body mass index; DLA, d-lactate; DAO, diamine oxidase; I-FABP, intestinal fatty acid binding proteins.

Most patients (128, 66.0%) were categorized in the AGI grade 1, and decreasing numbers of patients (25, 12.9% vs. 22, 11.3% vs. 19, 9.8%) were placed in grade 3, grade 2, and grade 4. The values of BMI were statistically different from AGI groups (*P *< .001), which from grade 1 to grade 4 were 25.4 (7.6) kg/m^2^, 25.8 (6.2) kg/m^2^, 30.2 (8.5) kg/m^2^, and 38.2 (6.6) kg/m^2^. Based on the values of BMI, there were 84 (43.3%) obese cases (BMI ≥28 kg/m^2^) and 110 (56.7%) nonobese cases. The distribution of AGI grading was significantly different between obese and nonobese cases (*P *< .001). The incidence of SAP was statistically higher in obese cases than that in nonobese cases (*x* ^2^ = 29.0, *P *< .001) (Table 2). The serum levels of DLA, DAO, and I-FABP could reflect the permeability of intestinal barrier in AP patients. The medians of DLA, DAO, and I-FABP were 31.8 (104.1) µg/L, 89.5 (252.3) µg/L, and 2.4 (17.7) µg/L, respectively. In the study, there was a moderate correlation between BMI and the serum levels of DLA (*R* = 0.5503, *P* < .001), DAO (*R* = 0.6399, *P* < .001), and I-FABP (*R* = 0.6524, *P* < .001) (Figure 4).

### Body Mass Index and Adverse Outcomes of Acute Pancreatitis

Meanwhile, AP-associated adverse outcomes were also related to the values of BMI. The BMI values were statistically higher in those patients developing MODS (30.4 [11.7] kg/m^2^ vs. 25.5 [7.0] kg/m^2^, *P* < .001), pancreatic necrosis (34.5 [8.3] kg/m^2^ vs. 25.8 [6.7] kg/m^2^, *P *< .001), receiving ICU admission (30.3 [8.5] kg/m^2^ vs. 25.7 [6.7] kg/m^2^, *P *< .001), and those who died (38.7 [12.1] kg/m^2^ vs. 26.5 [7.0] kg/m^2^, *P *< .001).

## Discussion

Acute pancreatitis is an inflammatory pancreatic disease, manifested by a spectrum of severity, ranging from MAP in the majority of patients to SAP in 10%-20% patients. Severe acute pancreatitis is associated with severe local and systemic complications and organ dysfunction.^13^ Majority of AP patients would lead to acute gastrointestinal malfunction characterized variously, such as with enterocyte injury, abnormal intestinal motility, and intestinal permeability damage. Secondary bacterial translocation from the gastrointestinal tract to the blood circulation system and to distant tissue and organs usually ensues, resulting in poorer prognosis.^14^ The mechanism of AP-associated gastrointestinal dysfunction is complicated. Several factors, like intestinal microcirculation disorder, excessive release of inflammatory mediators, ischemia-reperfusion injury, apoptosis, pyroptosis, intestinal nutrition deficiency, and dysbacteriosis are supposed to be involved.^15^ However, Ranson score, BISAP score, and the APACHE II score, the most frequently clinically utilized scoring systems of AP, could not accurately reflect gastrointestinal condition in early-stage AP patients.^16^ As a result, adverse outcomes in AP might be underestimated. Recognizing AP patients at risk of intestinal dysfunction can guide the initiation of proper clinical intervention and improve the prognosis.

Several literatures indicated that obesity was related to an increased inflammatory response and was a predicting factor for local and systemic complications in AP patients.^17,18^ Obesity had strong association with an increased pro-inflammatory condition which was supposed to be mediated by the metabolism of visceral adipose tissue and activity of obesity-associated cytokines. They also secreted higher inflammatory factors such as tumor necrosis factor-α (TNF-α), interleukin-6 (IL-6), and monocyte chemoattractant protein-1 (MCP-1). Although the definition of obesity varies, the most frequently used method for evaluating obesity is BMI. The value of BMI is calculated from a patient’s weight and height (kg/m^2^). Patient is categorized into 4 groups based on the World Health Organization classification: (1) underweight (<18.5 kg/m^2^), (2) normal weight (18.5-24.9 kg/m^2^), (3) overweight (25-29.9 kg/m^2^), and (4) obese (≥30 kg/m^2^).^19^ In our study, BMI levels were found to be higher in patients suffering from SAP compared to those suffering from MAP. Bedside index of severity in acute pancreatitis is a good scoring system, which has predictive capabilities for disease severity and mortality.^20^ Values of BMI were significantly increased from BISAP score 0 to 4. Similarly, adverse outcomes were also related to the values of BMI in the study. Furthermore, increased serum concentration of triglycerides in AP patients was independently related with persistent organ dysfunction and poor prognosis.^21^ In our study, the values of BMI in hyperlipidemic AP patients were statistically higher than those in other AP patients. Unsaturated fatty acids (UFA) generated from intra-pancreatic fat lipolysis worsened pancreatic inflammation and necrosis, and lipotoxicity mediated by UFA might have contributed to the higher incidence of MODS in obese AP patients.^22^

It is worth noting that obesity may damage intestinal mucosal barrier in early-stage AP. Mechanisms influencing gut permeability are disrupted in obesity, and this condition plays an aggravating role in several diseases, such as nonalcoholic fatty liver disease.^23^ However, the intestinal permeability in obesity complicated with AP had never been discussed. Ye et al^24^ demonstrated that obesity damaged intestinal integrity of AP rats and aggravated intestinal inflammatory injury. Exogenous leptin supplementation was in favor of anti-inflammation and improvement of intestinal mucosal barrier.^25^ In our study, values of BMI had moderate association with the serum concentrations of DLA, DAO, and I-FABP, which are the frequently utilized parameters to evaluate intestinal barrier permeability and intestinal flora translocation. However, DLA, DAO, and I-FABP could only reflect the gastrointestinal state at the micro-level. Acute gastrointestinal injury grading system was utilized to identify the gastrointestinal malfunction at the macro-level. Patients’ symptoms (e.g., vomiting, diarrhea, and hemorrhage), enteral feeding intolerance, and intra-abdominal hypertension were all supposed to evaluate the gastrointestinal injury. Body-mass index values were statistically different among AGI groups in the study, indicating that obesity related to intestinal dysfunction in early-stage AP.

There are several limitations in our study. First, this is single-central observational research with small sample size. Furthermore, the blood samples were not collected at the exactly same time after AP attack. Third, there is no control group of patients without AP. In the future, a multi-central controlled study on larger sample size will provide more evidence on the relationship between obesity and AP-associated intestinal dysfunction.

## Conclusions

This prospective study showed that obesity might contribute to increasing the severity of AP and aggravate subsequent intestinal injury in early-stage AP.

## Figures and Tables

**Table 1.** Clinical Features of AP Patients

Variable	
Male sex, n	121 (62.4%)
Age, years	57.2 ± 16.6
BMI, kg/m^2^	26.5 (7.0)
Etiology	
Gallstones, n	90 (46.4%)
Hypertriglyceridemia, n	48 (24.7%)
Alcohol, n	36 (18.6%)
Post-ERCP, n	10 (5.2%)
Others, n	10 (5.2%)
Severity	
MAP, n	116 (59.8%)
SAP, n	78 (40.2%)
BISAP score	1 (2)
CRP, mg/L	80.5 (60.0)
MODS, n	60 (30.9%)
Pancreatic necrosis, n	34 (17.5%)
Length of hospital stay, days	12 (7.5)
ICU admission, n	52 (26.8%)
Length of ICU stay, days	3.5 (5)
Mortality, n	10 (5.2%)

BMI, body-mass index; MAP, mild acute pancreatitis; SAP, severe acute pancreatitis; BISAP, bedside index of severity in acute pancreatitis; CRP, C-reactive protein; MODS, multiple organ dysfunction syndrome; ICU, intensive care unit.

**Table 2.** Relationship Between Obesity and AP-Associated Gastrointestinal Injury

Variable		Total, n	BMI, kg/m^2^		Obese Cases, n	Nonobese Cases, n	*P*
			*P*				
AGI score	AGI 1	128 (66.0%)	25.4 (7.6)	<.001	38	90	<.001
	AGI 2	22 (11.3%)	25.8 (6.2)		10	12	
	AGI 3	25 (12.9%)	30.2 (8.5)		17	8	
	AGI 4	19 (9.8%)	38.2 (6.6)		19	0	
Severity	SAP				52	26	<.001
	MAP				32	84	

BMI, body-mass index; SAP, severe acute pancreatitis; AGI, acute gastrointestinal injury.
